# The role of symbiotic fungi in the life cycle of *Gastrodia elata* Blume (Orchidaceae): a comprehensive review

**DOI:** 10.3389/fpls.2023.1309038

**Published:** 2024-01-08

**Authors:** Jia-Jia Liu, Xiao-Qi Yang, Zong-Yang Li, Jia-Yun Miao, Shi-Bo Li, Wen-Ping Zhang, Yi-Cen Lin, Lian-Bing Lin

**Affiliations:** ^1^ Faculty of Life Science and Technology, Kunming University of Science and Technology, Kunming, Yunnan, China; ^2^ Engineering Research Center for Replacement Technology of Feed Antibiotics of Yunnan College, Kunming, Yunnan, China; ^3^ Yunnan Key Laboratory of Gastrodia and Fungal Symbiotic Biology, Zhaotong University, Zhaotong, Yunnan, China; ^4^ Yunnan Senhao Fungi Industry Co., Ltd, Zhaotong, Yunnan, China

**Keywords:** Orchidaceae, Gastrodia elata, Mycena, Armillaria, fungi, mutualistic symbiosis

## Abstract

*Gastrodia elata* Blume, a fully mycoheterotrophic perennial plant of the family Orchidaceae, is a traditional Chinese herb with medicinal and edible value. Interestingly, *G. elata* requires symbiotic relationships with *Mycena* and *Armillaria* strains for seed germination and plant growth, respectively. However, there is no comprehensive summary of the symbiotic mechanism between fungi and *G. elata*. Here, the colonization and digestion of hyphae, the bidirectional exchange of nutrients, the adaptation of fungi and *G. elata* to symbiosis, and the role of microorganisms and secondary metabolites in the symbiotic relationship between fungi and *G. elata* are summarized. We comprehensively and deeply analyzed the mechanism of symbiosis between *G. elata* and fungi from three perspectives: morphology, nutrition, and molecules. The aim of this review was to enrich the understanding of the mutualistic symbiosis mechanisms between plants and fungi and lay a theoretical foundation for the ecological cultivation of *G. elata*.

## 1 Introduction

Orchidaceae is one of the largest plant families, comprising 750 genera and approximately 27 000 species (Dressler, 1981; Dressler, 1993; Chase et al., 2015). Orchids can be divided into three categories according to their different physiological characteristics. Fully photoautotrophic orchids obtain all necessary carbon from photosynthesis (Cameron et al., 2006). Partially mycoheterotrophic orchids require mycorrhizal fungi to stimulate seed germination and seedling growth and then develop green leaves and photosynthesize (Arditti, 1967). They obtain carbon from both fungi and photosynthesis (Rasmussen and Rasmussen, 2009). Fully mycoheterotrophic orchids are achlorophyllous and obtain their entire carbon supply from their associated mycorrhizal fungi (Leake, 1994). Over 99% of orchids live in nature as mycoheterotrophs (Merckx, 2013), of which more than 200 achlorophyllous orchid species from at least 25 lineages are full mycoheterotrophs (Leake, 1994; Merckx, 2013), including *Gastrodia elata* Blume. *G. elata*, known as “Tianma” in China, is a precious herbal medicine with high medicinal and nutritional value (Zheng et al., 2022) and is regarded as one of the most important medicinal herbs in Oriental countries (Sun et al., 2023).

*G. elata*, as a rootless, leafless, achlorophyllous, and fully mycoheterotrophic orchid, cannot produce nutrients by photosynthesis and can only survive symbiotically with fungi (Ho et al., 2021; Shan et al., 2021). It requires symbiotic interactions with *Mycena* and *Armillaria* strains to obtain nutrients for its complex life cycle. The seeds of *G. elata* are dust-like and lack nutritional reserves, which makes seed germination under natural conditions entirely dependent on *Mycena* (Park and Lee, 2013a). *Mycena* strains are responsible for the nutrient supply during seed germination, protocorm growth, and differentiation in the early stages of *G. elata* growth (Xu and Guo, 1989; Kim et al., 2006). *Armillaria* strains eventually replace *Mycena* strains as new symbionts of *G. elata* for tuber expansion, flowering, and fruit setting (Zhang and Li, 1980; Xu and Guo, 1989; Guo and Wang, 2001; Sekizaki et al., 2008; Park and Lee, 2013a). Different strains, as well as the growth rate and hyphal activity of symbiotic fungi, directly influence the quality and yield of *G. elata* (Guo and Wang, 2001; Wang et al., 2001a; Sun and Chen, 2003).

The coevolution of plants and fungi has been ongoing for over 400 million years, resulting in four main mycorrhizal types: ectomycorrhizas, arbuscular mycorrhizas, orchid mycorrhizas and ericoid mycorrhizas (Shi et al., 2023). The symbiosis between *G. elata* and fungi is one of the most unique mycorrhizas in orchids. Substantial knowledge on the interaction between *G. elata* and fungi, including structural and ultrastructural changes, nutrient transport, signal exchange, and genetic differences, has been gained through previous studies (Liu, 1981; Xu and Guo, 1991; Wang et al., 1997; Xu et al., 2001; Yuan et al., 2018; Cai et al., 2023). Different fungal strains affect the growth and quality of *G. elata* (Cha and Igarashi, 1995; Guo and Wang, 2001; Sekizaki et al., 2008; Guo et al., 2016), and fungal hyphae colonize *G. elata* by forming densely coiled structures called pelotons (Wang et al., 1997). The hyphae are digested to provide nutrients for *G. elata* and obtain nutrients from *G. elata* cells (Lan et al., 1994; Genre et al., 2020). Strigolactone was discovered to be an important signal that promotes the symbiotic relationship between *G. elata* and *Armillaria* (Yuan et al., 2018). The genomes of *G. elata* and *Armillaria* have recently been successively released, providing molecular evidence for their symbiosis (Xu et al., 2021; Cai et al., 2023).

In this review, we focus on the roles of *Mycena* and *Armillaria* in the life cycle of *G. elata* from the cellular scale to the ecosystem scale. Finally, the current understanding of the morphological, nutrient exchange, and molecular mechanisms underlying these symbiotic relationships is presented. This serves as both a theoretical guide for the planting and production of *G. elata* and a reference for the study of the symbiotic relationship between orchid mycorrhizae.

## 2 Overview of *G. elata* and its applications

### 2.1 Classification of *G. elata*

There are over 100 species in the genus *Gastrodia* (Orchidaceae), distributed in East Asia, Southeast Asia, and Oceania, with 36 species in China (Zhou et al., 2021b; Plant Plus of China, 2023; Plants of the World Online, 2023) (**Table 1**). Among them, *G. elata* is the most widely cultivated in China. There are six forms of *G. elata*: *G. elata* Bl. f. *glauca*, *G. elata* Bl. f. *viridi*s, *G. elata* Bl. f. *flavida, G. elata* Bl. f. *elata*, *G. elata* Bl. f. *pilifera*, and *G. elata* Bl. f. *alba* (Zhou and Chen, 1983). Among them, *G. elata* Bl. f. *glauca*, *G. elata* Bl. f. *viridi*s, *G. elata* Bl. f. *flavida, G. elata* Bl. f. *elata* are the four major forms that have been domesticated and cultivated. They have different inflorescence colors and tuber shapes. For example, the inflorescence colors of *G. elata* Bl. f. *glauca*, *G. elata* Bl. f. *viridi*s, *G. elata* Bl. f. *flavida*, and *G. elata* Bl. f. *elata* are dark, green, yellow, and red, respectively. In these subspecies, the mature tubers of *G. elata* Bl. f. *glauca* are the largest and have the highest contents of gastrodin (Wang et al., 2019).

**Table 1:** 36 Species of *Gastrodia* in China.

Serial Number	Chinese name	Latin Name	Time of first published	Protective grade
1	Tianma	*Gastrodia elata* Bl.	1856	Second-grade, VU
2	Wuhui tianma (Bai Tianma)	*Gastrodia albida* T. C. Hsu & C. M. Kuo	2011	CR, CITES Appendix II
3	Changguogeng Tianma (Mengla Tianma)	*Gastrodia albidoides* Y. H. Tan & T. C. Hsu	2012	
4	Yuan Tianma	*Gastrodia angusta* S. Chow & S. C. Chen	1983	Second-grade, EN, CITES Appendix II
5	Taiwan Tianma (Wuruihui Tianma)	*Gastrodia appendiculata* C.S.Leou & N.J.Chung	1991	CITES Appendix II
6	Bihua Tianma	*Gastrodia clausa* T. C. Hsu, S. W. Chung	2012	
7	Badai Tianma	*Gastrodia confusa* Honda & Tuyama	1939	VU, CITES Appendix II
8	Nibadai Tianma	*Gastrodia confusioides* T. C. Hsu, S. W. Chung & C. M. Kuo	2012	
9	Damingshan Tianma	*Gastrodia damingshanensis* A. Q. Hu & T. C. Hsu	2014	
10	Gaoshan Tianma	*Gastrodia dyeriana* King & Pantl.	1896	
11	Xia Tianma	*Gastrodia flavilabella* S. S. Ying	1984	CITES Appendix II
12	Zhezhu Tianma	*Gastrodia flexistyla* T. C. Hsu & C. M. Kuo	2010	
13	Chun Tianma	*Gastrodia fontinalis* T. P. Lin	1987	CITES Appendix II
14	Fujian Tianma	*Gastrodia fujianensis* Liang Ma, Xin Y. Chen & S. P. Chen	2019	
15	Xi Tianma	*Gastrodia gracilis* Blume	1856	CITES Appendix II
16	Nan Tianma	*Gastrodia javanica* (Blume) Lindl.	1840	CITES Appendix II
17	Gaoxiong Tianma	*Gastrodia kaoshiungensis* T. P. Lin	2018	
18	Hainan Tianma	*Gastrodia longitubularis* Q. W. Meng, X. Q. Song & Y. B. Luo	2008	
19	Menghai Tianma	*Gastrodia menghaiensis* Z. H. Tsi & S. C. Chen	1994	CITES Appendix II
20	Nantou Tianma	*Gastrodia nantoensis* T. C. Hsu & C. M. Kuo ex T. P. Lin	2016	
21	Beichatian Tianma	*Gastrodia peichatieniana* S.S.Ying	1987	CITES Appendix II
22	Dong Tianma	*Gastrodia pubilabiata* Sawa	1980	CITES Appendix II
23	Baidian Tianma	*Gastrodia punctata* Aver.	2006	
24	Qingyunshan Tianma	*Gastrodia qingyunshanensis* Jiu X. Huang, H. Xu & H. J. Yang	2021	VU
25	Hongbaoshi Tianma	*Gastrodia rubinea* T. P. Lin	2019	
26	Chaji Tianma	*Gastrodia shimizuana* Tuyama	1982	
27	Pingdong Tianma (Sushi Tianma)	*Gastrodia sui* C. S. Leou, T. C. Hsu & C. L. Yeh	2011	
28	Duanzhu Tianma	*Gastrodia theana* Aver.	2005	
29	You Tianma	*Gastrodia tuberculata* F. Y. Liu & S. C. Chen	1983	CITES Appendix II
30	Wulai Tianma	*Gastrodia uraiensis T.* C. Hsu & C. M. Kuo	2010	
31	Wuyishan Tianma	*Gastrodia wuyishanensis* D. M. Li & C. D. Liu	2007	CITES Appendix II
32	Huaping Tianma	*Gastrodia huapingensis* X.Y.Huang, A.Q.Hu & Yan Liu	2015	
33	Bawangling Tianma	*Gastrodia bawanglingensis* Z.H.Chen, Z.Y.Zhang & X.Q.Song	2023	
34	Changzhu Tianma	*Gastrodia longistyla* Q. Liu, J.D. Ya & X.H. Jin	2021	
35	Riben Tianma	*Gastrodia nipponica* (Honda) Tuyama	1939	
36	Fei Tianma	*Gastrodia callosa* J.J. Sm.	1931	

Second-grade, the second-grade protected plants according to the “List of National Key Protected Wild Plants (the second batch)”. CITES Appendix II, Appendix II of the Convention on International Trade in Endangered Species of Wild Fauna and Flora. VU, Vulnerable; EN, Endangered; CR, Critically Endangered (in the International Union for Conservation of Naturean Natural Resource).

However, wild *G. elata* is considered to possess higher medicinal value and is more popular and expensive than cultivated *G. elata*. As a result, wild *G. elata* is being overexploited and has been listed as a vulnerable species by the International Union for Conservation of Nature (IUCN) (Tsai et al., 2014; IUCN Red List, 2023). In China, *G. elata* has also been included in the second-grade protected plants according to the “List of National Key Protected Wild Plants (the second batch)” (List of National Key Protected Wild Plants, 2023).

### 2.2 The application value of *G. elata*

*G. elata* has many pharmacological effects (Wang et al., 2021b; Zhou et al., 2021a) and contains over 200 bioactive components and plant secondary metabolites, such as gastrodin (4-hydroxy methyl phenyl-β-D-glucopyranoside), gastrodigenin (p-hydroxybenzyl alcohol), p-hydroxybenzaldehyde, vanillin (4-hydroxy-3-methoxybenzaldehyde), parishin, *G. elata* polysaccharides, amino acids, and other compounds, which have been demonstrated to be the main components associated with the pharmacological activity of *G. elata* (Yu et al., 2005; Kim et al., 2007; Chen et al., 2011b). Among these, gastrodin and gastrodigenin are regarded as phytochemical indicators of *G. elata* in the Chinese pharmacopeia and are used in *G. elata* quality control (Sun et al., 2023).

*G. elata* has historically been used to treat headaches, vertigo, epilepsy, dizziness, paralysis, rheumatism, etc. (Kim et al., 2007). These effects were documented in *Shennong’s Classic of Materia Medica* (Shennong Bencaojing), which dates back to approximately 2000 years ago. Modern pharmacology research has shown that the tuber of *G. elata* has neuroprotective (Lu et al., 2022), anti-inflammatory, antidiabetic (Yang et al., 2018), antioxidative, antiepileptic, anticonvulsive, antipsychotic, anxiolytic, antidepressant (Gao et al., 2023; Huang et al., 2023), circulatory system modulating, memory-enhancing (Chen et al., 2011a; Chen et al., 2011c), cardiovascular disease ameliorating (Chen and Sheen, 2011), and other effects. Liu et al. (2018) summarized the mechanism by which *G. elata* acts on neurological diseases and psychiatric disorders, including modulating neurotransmitters, antioxidation, anti-inflammation, anti-apoptosis, suppressing microglial activation, regulating mitochondrial cascades, and upregulating neurotrophins. The efficacy of *G. elata* in treating cardiovascular diseases is mediated by its multitarget pharmacological properties, including reducing inflammation, inhibiting apoptosis, regulating autophagy, improving metabolism, inhibiting oxidative stress, and modulating the gut microbiota (Sun et al., 2023).

### 2.3 The life cycle of *G. elata*

**Figure 1:**
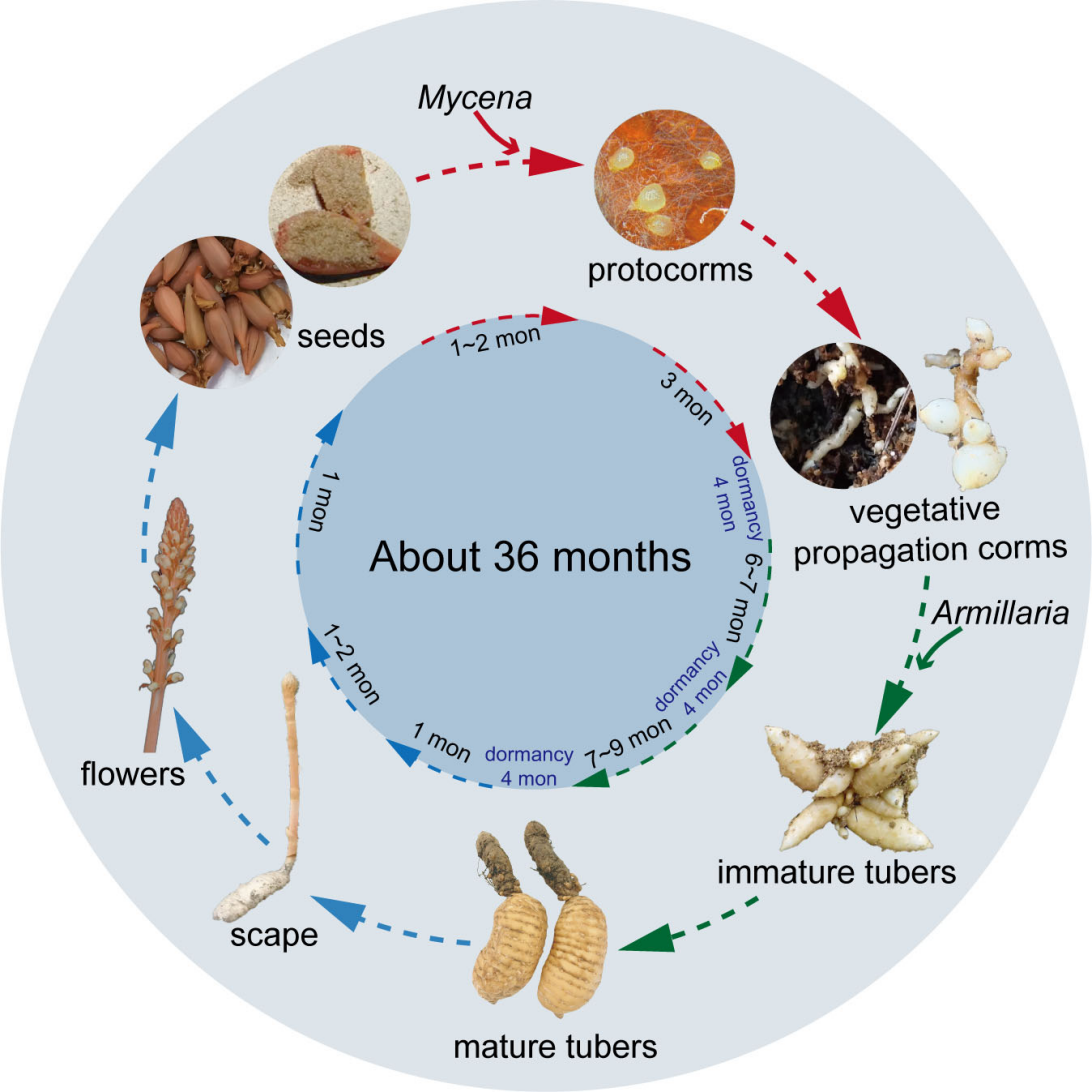
The life cycle of *Gastrodia elata*. *Mycena* is essential for the stage denoted by the red arrowhead, *Armillaria* is necessary for the stage depicted by the green shear head, and no fungi are required for the stage indicated by the blue shear head.

**Figure 2:**
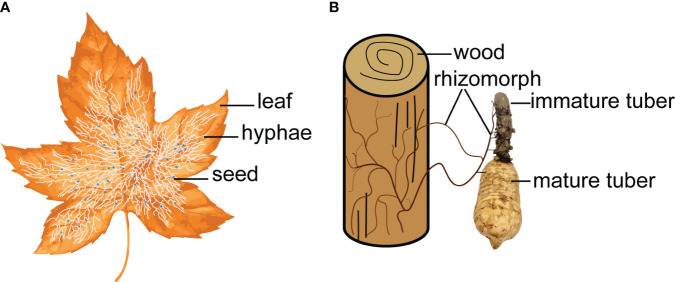
Symbiotic fungi act as a bridge to connect the nutrient exchange between leaf/wood and *G. elata*. **(A)**, Mycena. **(B)**, Armillaria.

Because of the complexity of the *G. elata* life cycle, vegetative growth and reproduction have long been a subject of biological speculation. Scientists did not know how vital symbiotic fungi were for *G. elata* growth until they fully comprehended the entire embryonic cycle (Zhou, 1981). The whole life cycle of *G. elata*, from seed germination to flowering plants, can take nearly three years and includes five stages: seed germination, protocorm growth and development, the first asexual reproduction to the formation of an immature tuber, the second asexual reproduction to the formation of a mature tuber, and the bolting, flowering and seed setting of the mature tuber (**Figure 1**). Among them, the first four stages are called the nutritional growth period of *G. elata*, and the last stage is the reproductive growth period. Generally, *G. elata* seeds are mixed with the *Mycena* strain and sown from June to August of the first year (Zhou, 1981; Xu et al., 1989). The seed coat ruptures as the embryo continues to grow during germination, releasing an oval-shaped tissue known as the protocorm. Protocorms progressively begin to form after approximately 20 days (Xu et al., 1989). The protocorm goes through cell division and tissue development, and at the top, a slender bud (some also produce branches) emerges. The meristem at the apex of the slender buds and branches continually expands, forming many bulbs with a diameter of approximately 2 mm. This form of *G. elata* is called the vegetative propagation corm (Zhou, 1981). The first asexual reproduction of vegetative propagation corms is initiated by exploiting *Armillaria*. When the bulb becomes a long strip with a diameter greater than 1 cm, it is considered an immature tuber. Immature tubers are tiny tubers of *G. elata* that cannot grow scapes and can be used for asexual reproduction (Xu et al., 1989); furthermore, *G. elata* spends the winter of that year as immature tubers. In the spring of the next year (approximately April), the immature tuber ends its dormancy for the second asexual reproduction. At this point, the immature tuber is similar to a mother tuber, developing a new tuber at its front end, which will form a mature tuber in autumn (**Figure 2B**). Immature tubers are sacrificed because their nutrients are exhausted by mature tubers. The flesh of mature tubers is thick and spherical, with an 8-20 cm body length. Notably, a protocorm can form multiple immature tubers, while an immature tuber can only produce one mature tuber. In the spring of the third year, the mature tubers end dormancy. Ultimately, a scape emerges from the mature tuber, and in summer, a flower develops from the scape to produce seeds. The nutrients for reproductive growth come entirely from mature tubers. The stored nutrients complete the whole process from bolting to seed maturation (Hsieh et al., 2022). More than 80% of the whole life cycle of *G. elata* is spent underground as a tuber (Yuan et al., 2018). Only the scape is exposed above the ground, and sunlight helps bees to carry out pollination (Sugiura, 2017). This is why wild *G. elata* is hard to find.

Currently, successful artificial cultivation and industrialization of *G. elata* can be achieved, primarily by mimicking wild cultivation beneath the forest floor. This lays the industrial foundation for the application of traditional Chinese medicine. However, there are many uncertain factors in the long life cycle, such as climate and wild animals, which increase the difficulty of cultivating *G. elata*. Recently, some scientists have tried to make the process less time-consuming by a tissue culture approach (Hsieh et al., 2022). During the life cycle, *G. elata* should be dormant for at least one winter; otherwise, the size and yield of the mature tubers will decrease. However, the mechanism is still unknown. We may be able to reduce the planting time of *G. elata* in the future by shortening its dormancy time.

## 3 Classification and function of symbiotic fungi

### 3.1 Mycena

It is estimated that a single plant of *G. elata* produces more than 2 million seeds; naturally, the seed germination rate is extremely low, and the yield is unstable (Zhou et al., 2005; Hsieh et al., 2022) because the seeds of *G. elata* are minute, with most containing an undifferentiated embryo that lacks a well-defined endosperm (Xu et al., 1990). Because of the lack of nutritional reserves, seed germination in nature entirely depends on *Mycena* strains, which provide nutrients needed for seed germination and protocorm development (Arditti, 1967; Xu et al., 1990; Xu and Guo, 1991; Kim et al., 2006; Dearnaley, 2007). Therefore, these fungi that can promote the germination of *G. elata* seeds are also known as germinating fungi.

**Figure 3:**
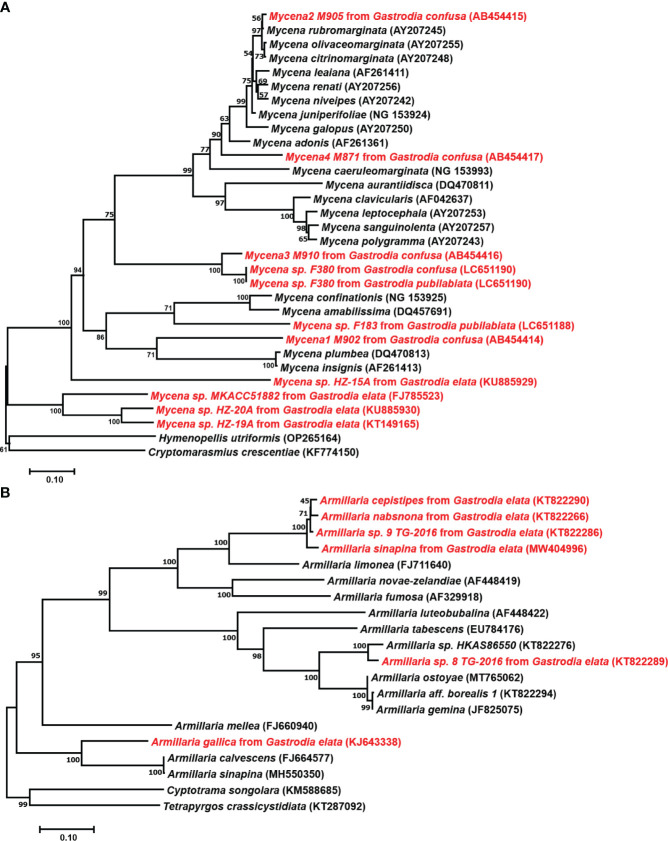
Phylogenetic positions of *Mycena* and *Armillaria*. **(A)**, Position of *Mycena* based on 28S LSU ribosomal RNA sequences. *Mycena* found in *Gastrodia confusa* by Ogura-Tsujita et al., 2009, *Gastrodia pubilabiata* by Kitahara et al., 2022, and *Gastrodia elata* from GenBank. **(B)**, Position of *Armillaria* based on ITS sequences. The GenBank ID are shown in parentheses, and the strains used for *Gastrodia* are highlighted in red. The tree was constructed using neighbor-joining with 1000 bootstrap replicates.

Germinating fungi of the genus *Mycena* belong to the family Mycenaceae of Basidiomycota and feed on dead trees and fallen leaves (Li et al., 2021). Xu et al. (1980) successfully discovered the sexual reproduction process of *G. elata* using the leaf fungal bed method, proving that under natural conditions, the sexual reproduction stage of *G. elata* can only germinate when nourished by germinating fungi. In 1989, protocorms were collected, and conventional tissue separation and monomer separation methods were used to isolate 12 strains that can effectively promote the germination of *G. elata* seeds (Xu and Guo, 1989). They also successfully induced growth of the fruiting body of the germinating fungus and identified it as *Mycena osmundicola* by morphological and microscopic observation and enzyme ester isozyme analysis. To study the existence of germinating fungi in the distribution areas of wild *G. elata*, (Xu et al., 2001) collected deciduous humus soil from the original site to sow *G. elata* seeds and isolated 11 strains from germinated protocorms. After mixing and sowing *G. elata* seeds, they obtained three fungi that had a promoting effect on germination. To study the diversity of fungal strains for seed germination of *G. elata*, scientists isolated 132 strains from the roots of 45 orchid plants and found that *Mycena dendrobii* (Guo et al., 1999), *Mycena anoectochila* (Xu et al., 2001), and *Mycena orchidicola* (Fan et al., 1996) could promote seed germination of *G. elata*. Guo et al. (1999) isolated *M. dendrobii* from wild *Dendrobium densiflorum* and conducted symbiotic germination experiments with seeds of 12 orchid species, and the results showed that the fungus could promote the growth of *G. elata* and *D. densiflorum.* From this, we speculate that germinating fungi can not only promote the germination of *G. elata* seeds but also coexist with other orchid plants, indicating that germinating fungi have a wide range of applications. In existing studies, most mycorrhizal *Mycena* were isolated from various members of the Orchidaceae or protocorms of *G. elata*. Species of *Mycena* with tiny basidiomata are abundant, which complicates identification without basidiomata solely based on the few reliable DNA sequences in GenBank (**Figure 3A**). Currently, only four species (*M. osmundicola*, *M. orchidicola*, *M. dendrobii*, and *M. anoectochila*) are known to be able to form basidiomata in cultivation and have thus been successfully identified (Xu and Guo, 1989; Fan et al., 1996; Guo et al., 1997; Guo et al., 1999). Therefore, these four species have become commonly used fungi for seed germination of *G. elata* in China (Guo and Wang, 2001; Park and Lee, 2013a; Pan et al., 2015; Kitahara et al., 2022).

### 3.2 Armillaria

The genus *Armillaria* belongs to the family Physalacriaceae of Basidiomycota (Sipos et al., 2018)*. Armillaria* has a strong ability to degrade cellulose and lignin, making it the causative agent of forest root rot (Sipos et al., 2017). However, *Armillaria* is essential for the growth of *G. elata*. Mycorrhizal symbiosis between *Armillaria* and *G. elata* was first described by Kusano, 1911. In 1965, Xu successfully cultivated *G. elata* for the first time by using wood with *Armillaria* (Xu, 2013), and he summarized a set of asexual propagation and cultivation techniques suitable for the large-scale production of *G. elata*.

The classification and identification of *Armillaria* is relatively complex. The sexual reproduction of *Armillaria* makes species identification based on the morphological characteristics of fruiting bodies reasonable. However, the macroscopic and microscopic characteristics of fruiting bodies overlap widely among related species (Antonín et al., 2009; Park et al., 2018). After the discovery of the tetrapolar heterothallic coordination mechanism of *Armillaria* and the difference in colony morphology between haploid and diploid fungi, this method was widely used to identify fungal species. However, single-spore (haploid) isolates must be available for mating assays. This restricts their value in identifying samples collected as rhizomorphs, which are frequently connected to *G. elata* tubers (Guo et al., 2016). Recent years have seen a rise in the use of molecular data, notably DNA sequence data, to identify fungal species (Cai et al., 2011). Coetzee et al. (2000) clarified the phylogenetic relationships among biological species of *Armillaria* from China based on the sequences from tef1-alpha and IGS-1 genes and resolved four main phylogenetic groups, namely, the “*Armillaria mellea*”, “*Armillaria ostoyae*”, “*Armillaria tabescens*”, and “*Armillaria gallica*” clusters. Guo et al. (2016) phylogenetically analyzed Chinese *Armillaria* samples using the sequences of the internal transcribed spacer region, translation elongation factor-1 alpha gene and beta-tubulin gene and revealed at least 15 phylogenetic lineages of *Armillaria* from China, in which 7 phylogenetic lineages of *Armillaria* were used for the cultivation of *G. elata*. They also found that *G. elata f. glauca* and *G. elata f. elata* form symbiotic relationships with various phylogenetic lineages of *Armillaria*.

In the *G. elata* growth stage, *Armillaria* is the only nutrient source, and the growth rate, activity, and other characteristics of the strain directly affect the quality and yield of *G. elata*. Hyphae are the nutrient organ of *Armillaria*. The rhizomorph is an adaptive metamorphosis of hyphae that occurs under adverse environmental conditions or in the later stages of growth. It mainly plays a role in transporting nutrients, water, and oxygen while constantly proliferating, extending, and searching for new nutritional sources (Wong et al., 2020). However, few *Armillaria* species can benefit the growth of *G. elata* (**Figure 3B**). *A. mellea*, *A. gallica, Armillaria sinapina, Armillaria singula, Armillaria nabsnona*, etc., are widely used in the cultivation of *G. elata* (Zhang and Li, 1980; Xu and Guo, 1989; Guo and Wang, 2001; Sekizaki et al., 2008). Additionally, because *Armillaria* species degeneration occurs during multigenerational reproduction because of the unstable compatibility of foreign strains with *G. elata* in the primary production area, a focus of study has been on isolating and identifying more and better *Armillaria* strains to increase strain resources.

## 4 Mechanism of interaction between symbiotic fungi and *G. elata*

### 4.1 The process of fungal colonization and digestion

**Figure 4:**
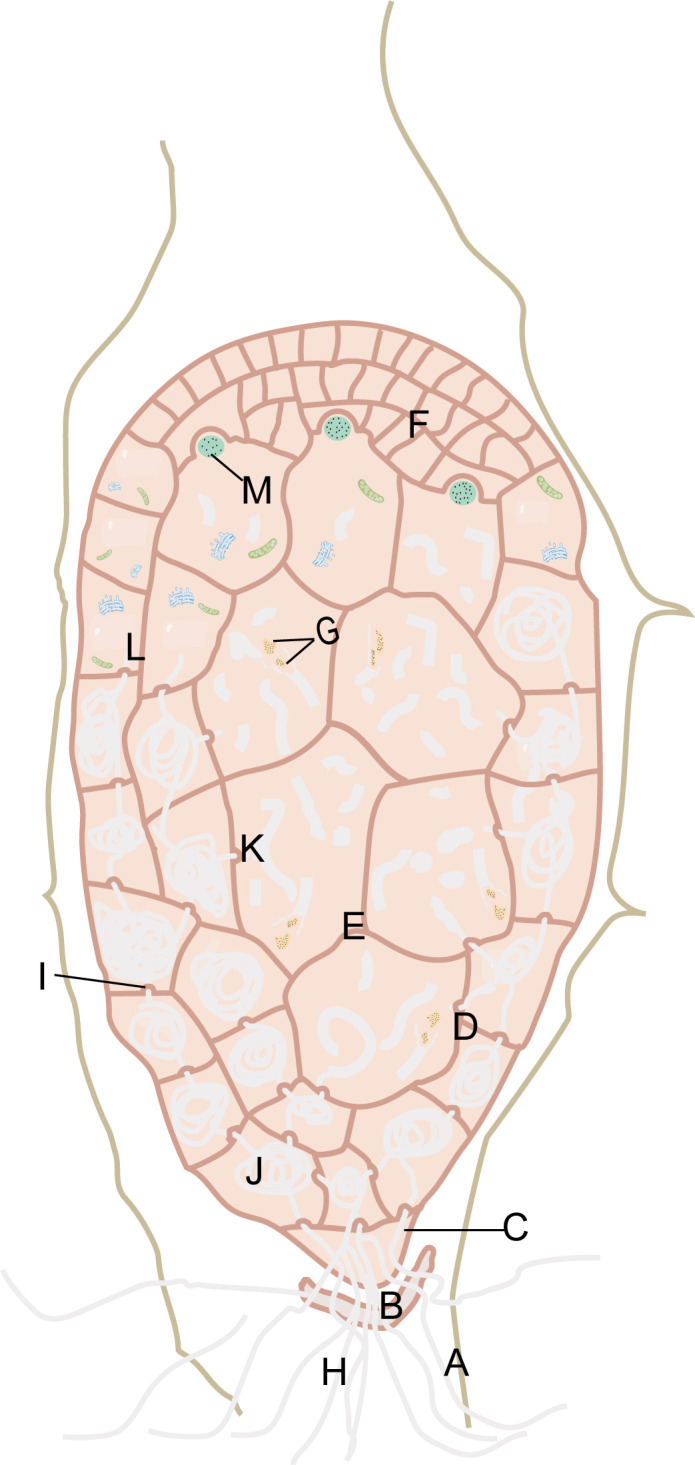
*Mycena* invade the seeds of *G*. *elata.* Seed-coat layer **(A)**. Suspensor remnant **(B)**. Stipe cell **(C)**. Peloton cells **(D)**. Digestive cells **(E)**. Meristematic cells **(F)**. Released cytoplasm and organelles of the hyphae **(G)**. Hyphae **(H)**. Papillary protrusion **(I)**. Peloton **(J)**. Hyphal fragments **(K)**. The proembryo cells in the initial stage of hyphal invasion **(L)**. Digested hyphae **(M)**.

Previous studies on the symbiotic germination of *G. elata* seeds and *Mycena* offered extensive information on the changes in structure and ultrastructure (Peterson and Currah, 1990; Xu, 1990; Uetake et al., 1997; Fan, 1998; Fan et al., 1999a; Fan et al., 1999b; Fan et al., 2002; Chen et al., 2014; Li et al., 2020b). *Mycena* invades *G. elata* seeds, first penetrating the seed coat layer and then moving through the suspensor remnant, stipe cell, peloton cells, and digestive cells (**Figure 4**). Mature seeds of *G. elata* have an oval-shaped proembryo surrounded by a thin seed coat layer (Xu and Guo, 1989). The proembryo consists of stipe cells, proembryo cells, and meristematic cells. A layer of suspensor remnant rich in nutrients such as polysaccharides is attached to the periphery of the stipe cell, resembling a gelatinous cell structure, and is a vestige of stipe cells that degenerate during embryonic development (Xu and Fan, 2001). Hyphae of *Mycena* can invade from any cell in the seed coat, accumulate in the suspensor remnant, and then enter the stipe cell (Fan et al., 1999b). The stipe cell is the only pathway for hyphae to invade the proembryo (Li et al., 2020b). When hyphae invade the proembryo from the stipe cell, the proembryo will differentiate into peloton cells and digestive cells. Proembryo cells with a peloton are called peloton cells (Fan et al., 1999a), while digestive cells are large proembryo cells that have the capacity to break down hyphae (Fan et al., 1999a). At the initial stage when proembryo cells are invaded by hyphae, organelles such as mitochondria, endoplasmic reticulum and vacuoles may play a role in the digestion of hyphae (Xu and Fan, 2001). Gradually dominant hyphae can utilize nutrients from embryonic cells for reproduction. The cytoplasm and organelles of the proembryo cells will no longer exist, and the hyphae will be full of cells and form a peloton (Xu and Fan, 2001). In the peloton cells, hyphae are complete in structure, rich in contents, vigorous, and sometimes vacuolated. Digestive cells are the key sites of hyphal digestion, in which hyphae expand rapidly, integrity is destroyed, cytoplasm and organelles are all released, and hyphae decay. After leaving the peloton cells, the hyphae extend inward into larger cells, where they no longer form hyphae and are gradually digested. Digested hyphae reach apical meristematic cells through intercellular transmission (Lan et al., 2002). The meristematic cells obtain nutrients and undergo vigorous division, and the embryo expands, breaking through the seed coat and germinating, forming protocorms. The protocorm differentiates into vegetative propagation corm and vascular tissue. The hyphae invading the embryo cells continue to infect the top of the protocorm along cortical cells, but when they are near the top of the protocorm and below the meristematic cells, the hyphae no longer continue to infect upward (Li et al., 2020b). Regardless of whether the protocorm has a nutritional relationship with *Armillaria*, it can undergo asexual reproduction to form a vegetative propagation corm. The vegetative propagation corm needs nutrients from *Armillaria* as soon as possible; otherwise, it will die due to nutrient depletion. Li et al. (2020b) found that there was a tubular endocytic network attached to the lysed hyphae in cortical cells, so they concluded that the hyphae were digested by endocytosis. These discoveries provide crucial clues for the symbiotic seed germination of *G. elata* with *Mycena*.

**Figure 5:**
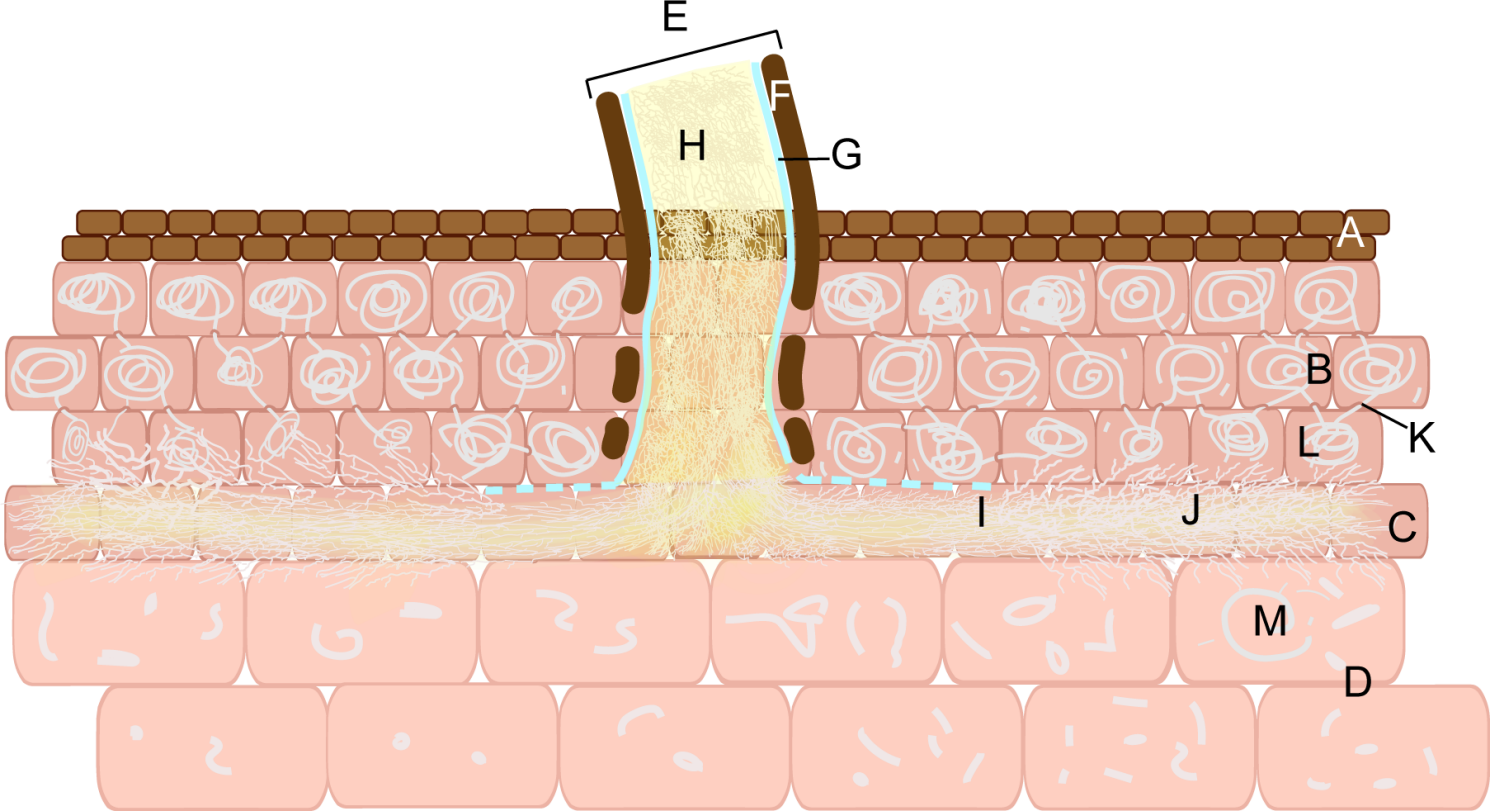
*Armillaria* invades the vegetative propagation corm of *G*. *elata.* Epidermal cells **(A)**. Peloton cells **(B)**. Susceptible fungal cell **(C)**. Digestive cells **(D)**. Rhizomorph **(E)**. Outer sheath **(F)**. Membrane **(G)**. Hyphae **(H)**. The hyphal channel **(I)**. Hyphal flow **(J)**. Papillary protrusion **(K)**. Peloton **(L)**. Hyphal fragments **(M)**.

*Armillaria* invades the vegetative propagation corm of *G. elata* in turn through the susceptible fungal cells, the hyphal channel or the hyphal flow, the peloton cells, the hyphal flow, and the digestive cells (**Figure 5**). The cell layer of the vegetative propagation corm is, from outside to inside, the epidermis, peloton cells in the cortex, the hyphal channel or hyphal flow of susceptible fungal cells, and digestive cells in the endodermis (Xu and Guo, 2000). *Armillaria* must first develop rhizomorphs attached to the epidermis of *G. elata* before invading it. The hyphae in the rhizomorph penetrate the epidermal cells of *G. elata* with mechanical force and directly enter a layer of cortical cells outside the endodermis (Xu, 2001). This layer of cells is known as susceptible fungal cells by certain scientists (Wang et al., 1997; Xu, 2001; Xu and Fan, 2001). During the colonization process, the outer sheath and cortex of the rhizomorph are gradually dissolved, leaving only a layer of membrane surrounding the hyphae but still maintaining the morphology of the rhizomorph, which is called the hyphal channel (Wang et al., 1997; Xu, 2001; Xu and Fan, 2001). The hyphae of *Armillaria* break through the membrane; that is, it loses the form of the rhizomorph and spreads into the cells of the new susceptible fungal cells in the form of hyphae. The bundle-shaped hyphae are called hyphal flow (Wang et al., 1997; Xu, 2001; Xu and Fan, 2001). At this point, the hyphae can nourish themselves with the protoplasm of susceptible fungal cells and colonize the surrounding areas. The hyphae invade the digestive cells inward and the peloton cells outward, with the hyphae flow as the center. The cell walls of the colonized cells exhibit papillary protrusions (Wang et al., 1997; Xu, 2001; Xu and Fan, 2001). After penetrating the papillary protrusions, the hyphae reach digestive cells, where *Armillaria* is digested (Xu and Mu, 1990). Within cortical cells, hyphae are enveloped by vesicles generated by the protoplasm of the cells. These vesicles twist and coil the hyphae into pelotons, facilitating their gradual division into fragments for subsequent digestion and absorption (Wang et al., 1997; Xu, 2001; Xu and Fan, 2001). The hyphal fragments are released into digestive cells via hyphal flow. Additionally, pelotons have the capacity to breach cell walls and enter adjacent cortical cells (Xu and Mu, 1990). In digestive cells, *Armillaria* is ultimately digested and absorbed, and viable hyphae cease to exist.

### 4.2 Nutrient acquisition in symbiotic relationships

In nature, some mycoheterotrophic orchids are associated with ectomycorrhizal fungi and form tripartite symbioses between trees, mycobionts and orchids (McKendrick et al., 2000). The *G. elata* growth process is a ternary germination and cropping system (Yuan et al., 2020), which depends on a symbiotic relationship with *Mycena* and *Armillaria.* Symbiotic fungi decay leaves or wood to obtain nutrients for their own growth and provide nutrients for *G. elata* due to colonization and digestion (Suetsugu et al., 2020). Symbiotic fungi act as a bridge to connect the nutrient exchange between leaf/wood and *G. elata* (**Figure 2**). Scientists have investigated the effects of different wood as a substrate on the size and ergothioneine concentrations in *G. elata* (Rong and Cai, 2010). Park and Lee (2013b) investigated 14 tree species and suggested that the use of *Ulmus davidiana* might increase the production of *G. elata* tubers.

Symbiotic fungi not only provide nutrients for *G. elata* but also obtain nutrients from it (Xu and Guo, 1989; Lan et al., 1994; Xu and Guo, 2000; Lan et al., 2002). From the time the proembryo cells are initially able to digest hyphae to the point when they are colonized by hyphae is the stage of mutual benefit in the symbiotic relationship between *Mycena* and *G. elata*. Lan et al. (2002) labeled *M. osmundicola* with ^3^H-glucose, and the seeds of *G. elata* were sown on the saprophytic leaves of labeled *M. osmundicola*. They discovered that many developing silver grains were also found in the newly formed vascular tissue of vegetative propagation corms, indicating that *M. osmundicola* not only provided nutrients during seed germination and protocorm formation but also needed nutrients from germinating fungi during vegetative propagative corm differentiation and growth. In the symbiotic nutrient chain between *G. elata* and *Armillaria*, during hyphal flow, *Armillaria* can utilize the protoplasm of susceptible fungal cells, which is a favorable stage for *Armillaria*. Lan et al. (1994) demonstrated this by using ^3^H-glucose to label *G. elata* with the pouring method. In cortical cells, *Armillaria* can utilize the nutrients of cortical cells for division and growth and can invade new cortical cells via a peloton cell. Moreover, some hyphae are also digested by *G. elata* cortical cells, which is beneficial for the growth of both *G. elata* and *Armillaria*. Cortical cells are the site of symbiosis between *G. elata* and *Armillaria*. In digestive cells, the complete digestion and absorption of hyphae is a favorable stage for *G. elata*.

The carbon source has been revealed to be critical to establish optimal symbiosis (Kiers et al., 2011; Hennion et al., 2019). The nutrient exchange between plants and mycorrhizal fungi is that plants provide photosynthetically fixed C to symbiotic fungi, and they benefit from fungi by absorbing mineral nutrients, such as N and P (Bücking and Kafle, 2015; Jacquemyn et al., 2015; Wang et al., 2017). Cameron et al. (2006) demonstrated for the first time mutualism in orchid mycorrhizae, bidirectional transfer of C between a green orchid and its fungal symbiont, and a fungus-dependent pathway for organic N acquisition by an orchid. However, (Fochi et al., 2017)investigated the expression of fungal and plant nitrogen (N) transport and assimilation genes in mycorrhizas formed between the fungus *Tulasnella calospora* and the achlorophyllous protocorms of the photosynthetic orchid *Serapias vomeracea*. Their research suggested, for the first time, that nutrients flow back to the fungal partner from the nonphotosynthetic orchid host (Dearnaley and Cameron, 2017). Yeh et al. (2019) proposed that cells of nonphotosynthetic orchids export ammonium (NH_4_^+^) to their fungal partners and receive N, P and C for germination and growth. In addition, decayed pelotons can also release N, P and C to nonphotosynthetic orchids when pelotons are digested (Bougoure et al., 2014). This result is largely consistent with the nutrient exchange between *G. elata* and symbiotic fungi. The nonphotosynthetic orchid *G. elata* completely replaces its photosynthetic capacity by taking up C from symbiotic fungi (Suetsugu et al., 2020). The symbiotic fungi *Mycena* and *Armillaria* obtain C through parasitism of fallen leaves or wood, providing all C sources to maintain the germination and growth of *G. elata* (Kikuchi et al., 2008a; Kikuchi et al., 2008b). Because the cell walls of fungi are mainly composed of glucan and chitin (Ruiz-Herrera and Ortiz-Castellanos, 2019; Chen et al., 2020), the digestion of hyphae may provide a large amount of organic C and N for *G. elata*. Moreover, high levels of sucrose accumulate in *G. elata* tubers at all stages, indicating that sucrose may be the main form of carbohydrates transported to *G. elata* at the symbiotic interface (Ho et al., 2021). Symbiotic cells are the main sites for extracellular sucrose exchange at the heterotrophic interface of fungi. Sugar transporters have been identified that are located on contiguous plant and fungal cells, and these transporters may regulate sugar exchange, ensuring benefits for both partners in this symbiotic relationship (Hennion et al., 2019). The sucrose transporter gene SUT4 in *G. elata* was shown to mediate sucrose import at the symbiotic interface for carbon allocation of *Armillaria*-colonized juvenile tubers (Ho et al., 2021). Based on the amplification of the gene encoding trehalase in the genome of *Gastrodia menghaiensis*, a species closely related to *G. elata*, (Jiang et al., 2022) proposed that it may have evolved the ability to use trehalose as its organic carbon source. The absence of nitrate transporters and the increase in the number of urease genes indicate that the absorption of nitrogen by *G. menghaiensis* mainly occurs in the form of ammonium. While most raw nutrients primarily originate from fungi, the highly expressed genes for fatty acid and ammonium root transporters indicate that fungi obtain nutrients from *G. menghaiensis*. *G. elata* may share some features with *G. menghaiensis*, however, further research is needed. Arginases can hydrolyze arginine acid in hyphae to urea, which is further hydrolyzed to ammonium and carbonic acid by ureases (Witte et al., 2005). The number of genes encoding ureases in *G. elata* is sharply increased, indicating that urea metabolism may be an important source of N for *G. elata* (Yuan et al., 2018).

Through experimental studies, the penetration of *Armillaria* into *G. elata* is divided into two forms (Xu and Guo, 2000; Morrison, 2004). One is normal physiological colonization. *G. elata* induces *Armillaria* colonization by secreting specific chemicals, such as strigolactone (Hua et al., 2024), and then secretes enzymes to digest *Armillaria* for energy. The more *Armillaria* colonizes, the more energy *G. elata* acquires and the more quickly it grows. The other is pathological infection; *Armillaria* will penetrate the digestive layer of the mother tuber (immature tuber), infiltrate the stele layer, and subsequently invade the new tuber along the vascular bundle, resulting in the decay of the new tuber. However, this rarely happens. When *Armillaria*’s nutritional supply is insufficient and *G. elata*’s development or resistance declines, *Armillaria* will use the nutrients in *G. elata* to grow (Xu and Guo, 2000). Therefore, we speculate that if *G. elata* has strong inducibility to the corresponding *Armillaria* and the invasion of *Armillaria* is weak, *G. elata* grows normally. In contrast, if inducibility is weak and invasiveness is strong, *G. elata* will be consumed. Therefore, only the combination of *G. elata* with strong inducibility and *Armillaria* with weak invasion ability can allow *G. elata* to obtain nutrients through corresponding strategies for maintenance.

### 4.3 Adaptation of fungi to symbiosis

The increased secretion of some enzymes by fungi is beneficial for establishing symbiotic relationships. The seed coat of *G. elata* is composed of only lignin (Li et al., 2016), and the lignin-degrading ability of the germination-promoting fungus *Mycena* is the potentially key to their symbiosis. Manganese peroxidase is a fungal lignin-modifying enzyme, and when *Mycena* breaks through the seed coats of *G. elata*, manganese peroxidase and laccase are responsible for the degradation of lignin (Manavalan et al., 2015). Ren et al. (2021) conducted transcriptome analysis and found that the upregulation of manganese-dependent peroxidase short genes was conducive to the invasion of *G. elata* seeds by hyphae. In addition, the expression of laccase genes was significantly upregulated to produce more laccase for degrading the lignin seed coat. Compared with nonsymbiotic *Armillaria*, *Armillaria* that were symbiotic with *G. elata* had more glycoside hydrolases, carbohydrate-binding modules, and glycosyl transferases (Zhan et al., 2020; Cai et al., 2023). These enzymes contribute to the degradation of cell walls, fungal colonization and secondary metabolic synthesis, (Deng et al., 2021; Liu et al., 2021) which may contribute to the successful establishment of symbiosis between *Armillaria* and *G. elata*. Virulence attenuation is related to the enhanced adaptability of fungi to *G. elata*. Once symbiosis is established, *G. elata* begins to grow with increased biological activity, while the fungi are restricted (Fan et al., 1999a). Ren et al. (2021) showed this through comparative transcriptome analysis of seed symbiotic *Mycena* hyphae and pure cultured hyphae, and furthermore, they sequenced and analyzed the genome of *Mycena* and found that 5024 genes were annotated in the pathogen-host interactions database, among which more than half were linked to reduced virulence and loss of pathogenicity. Zhan et al. (2020) assembled a draft genomic sequence of *A. gallica* 012m and found that the gene families related to the pathogenicity/saprophytic phase, including hydrophobins, carbohydrate active enzyme AA3, and cytochrome P450 monooxygenases, had significantly contracted in *A. gallica* 012m, which might be beneficial for *G. elata* to reduce injury. They also found, through genome-guided analysis, that rhizomorphs exhibit higher infectivity compared to vegetative mycelia. This characteristic aids *G. elata* in nutrient acquisition, as rhizomorphs continually colonize *G. elata*’s nutritional stems and generate hyphae that *G. elata* can subsequently digest. Guo et al. (2016) revealed at least 15 phylogenetic lineages in China through the phylogenetic analysis of *Armillaria*, of which 7 species that are less virulent and aggressive or preferentially saprotrophic are related to cultivated *G. elata*. Virulence experiments demonstrated that *A. mellea* has a greater or equal virulence than *A. ostoyae*, *A. ostoyae* has a greater virulence than *A. gallica* and *A. cepistipes*, and *A. tabescens* has the weakest virulence among those five species (Gregory, 1985; Morrison, 2004; Caballero et al., 2022). The reason is that the saprophytic colonization scores of *Armillaria* with monopodially branched rhizomorphs are significantly higher than those of dichotomously branched species, while the dichotomously branched species are more aggressive than monopodially branched species (Morrison, 2004) (**Table 2**). Therefore, we speculate that *Armillaria* with monopodially branched rhizomorphs is more suitable for cultivating *G. elata*.

**Table 2:** Rhizomorph growth habit of some *Armillaria* species (Morrison, 2004).

Armillaria species	Rhizomorph growth habit
*A. gallica*	Monopodial
*A. cepistipes*	Monopodial
*A. gemina*	Monopodial
*A. sinapina*	Monopodial
*A. calvescens*	Monopodial
*A. nabsnona*	Monopodial
*A. mellea*	Dichotomous
*A. ostoyae*	Dichotomous
*A. borealis*	Dichotomous
*A. luteobubalina*	Dichotomous
*A. fumosa*	Dichotomous
*A. hinnulea*	Dichotomous
*A. novae-zelandiae*	Dichotomous
*A. limonea*	Dichotomous

### 4.4 Response of *G. elata* to fungi

The evolutionary adaptation of *G. elata* to the mycoheterotrophic lifestyle was critically dependent on gene loss. The genome of *G. elata* has been continuously reported in recent years, offering insights into how *G. elata* adapted to heterotrophy in *Mycena* and *Armillaria*. In *G. elata*, genes important in the control of flowering time, the circadian clock, nutrient absorption, immunity, growth of the roots and leaves, and photosynthesis were all severely lost (Yuan et al., 2018; Park et al., 2020; Xu et al., 2021; Bae et al., 2022).

The expansion of many gene families in *G. elata* is additional evidence for adaptation to the mycoheterotrophic lifestyle of fungi. Phylogenetic analysis showed that the number of genes of *G. elata* concerned with mycorrhizal association was significantly expanded (Bae et al., 2022; Jiang et al., 2022). These phenomena represent evolutionary events and may be the result of *G. elata* adapting to a heterotrophic lifestyle in the presence of *Armillaria* (Yuan et al., 2018; Bae et al., 2022). Yuan et al. (2018) found that the increase in the number of genes encoding carotenoid cleavage dioxygenases and ABC transporters indicated that *G. elata* has a strengthened ability to interact with *Armillaria* to improve the efficiency of establishing symbiotic relationships. Genes involved in the Ca^2+^ spiking process have been shown to regulate the colonization of plants by fungi and are found in large quantities in *G. elata*. Some glycoside hydrolases from gene families were highly expressed in the cortex layer of *G. elata*, which supports the view that the hyphae of *Armillaria* are digested in the digestive cells of *G. elata*. Strigolactone is a plant hormone that has been proven to have branch-inducing effects in *Armillaria* (Yuan et al., 2018; Favre-Godal et al., 2020). The number of key genes for the biosynthesis and secretion of strigolactone has grown in *G. elata* (Yuan et al., 2018). Jiang et al. (2022) found that 36 beta-glucosidase genes and 4 glycoside hydrolase family 18 chitinases in *G. menghaiensis* may be involved in the degradation of the fungal cell wall to provide nutrients for *G. menghaiensis.*

Gene contraction in *G. elata* is a characteristic facilitating adaptation to the mycoheterotrophic lifestyle. *G. elata* seed germination is hampered not only by insufficient nutritional reserves and an impermeable seed coat but also by the presence of seed germination inhibitors such as phenolics and abscisic acid (Van Waes and Debergh, 1986; Ren et al., 2021). The downregulation of 9-cis-epoxycarotenoid dioxygenase (NCED-2) expression in *G. elata* reduces abscisic acid production, thereby alleviating abscisic acid’s suppression of seed germination. Additionally, a significantly downregulated receptor protein (PYL12-like) can hinder abscisic acid signaling and thereby break seed dormancy (Ren et al., 2021).

*G. elata* must fend against pathogen assaults despite being fully dependent on symbiotic fungi for survival. Significant defensive reactions are induced by fungal colonization in *G. elata*, which possesses genetic, pharmacological, and physical defenses against fungi. *Armillaria* can only colonize vegetative propagation corms and immature tubers (mother tubers). Newborn tubers can resist colonization by *Armillaria*. From the perspective of *G. elata*, this is because there is an isolation area without nutrient reserves at the contact between the vegetative propagation corm and the new tuber, which limits the spread of *Armillaria* along the cortical cells to invade the new tubers. The cell wall of the mother tuber near the new tuber thickens and becomes corky. Then, a fracture layer will form at the bottom of the new tuber. The corked and thickened cell wall is a physical defense structure of *G. elata* against *Armillaria* infection (Fan et al., 1999a).

The epidermal cells of vegetative propagation corms are initially digested by the matching enzymes released by *Armillaria* when they penetrate. *G. elata* causes cortical cells to create numerous hydrolases as a form of defense after learning that epidermal cells have penetrated. Hydrolase breaks hyphae down into tiny molecules that *G. elata* may consume, supplying it with ongoing nutrients for growth. (Wang and Xu, 1993; Fan et al., 1999a) Additionally, *Armillaria* has the ability to induce *G. elata* to produce a set of defense proteins (Yuan et al., 2020). The monocot mannose binding lectin antifungal protein family has been proven to inhibit fungal growth in *G. elata* (Xu et al., 1998; Wang et al., 2001b; Nagel et al., 2008). More than 80% of the gastrodia antifungal protein genes in *G. elata* are highly expressed during the growth stage before establishing a stable symbiotic relationship with *Armillaria* (Yuan et al., 2018). In addition, *G. elata* can transport S-(p-HA)-glutathione phytoalexin to *Armillaria* and prevent its excessive growth. Defense proteins and phytoalexins are chemical defenses of *G. elata* against *Armillaria* infection (Yuan et al., 2018).

*G. elata* also retains some defense-related genes. Zeng et al. (2017) used transcriptome approaches to identify 1750 differentially expressed genes between *G. elata* seeds and protocorms. Most of these differentially expressed genes were presumably involved in plant defense, molecular signaling, secondary metabolism, and energy metabolism. Zeng et al. (2018) compared the proteomes of the early and late stages of protocorms. Among them, defense genes (e.g., pathogenesis-/wound-related proteins, peroxidases, and serine/threonine-protein kinase) were highly expressed in late-stage protocorms, suggesting that fungal colonization triggered significant defense responses in *G. elata*. The *G. menghaiensis* genome contains 28 terpene synthase genes, which defend against pathogens (Zeng et al., 2017). The *G. menghaiensis* genome contains 65 R genes (resistance), which are important components of the plant defense system (Ren et al., 2021).

### 4.5 Microbes can affect the symbiosis of *G. elata* and *Armillaria*

Microbes and their secondary metabolites are also believed to affect the symbiosis of fungi and *G. elata*. The fungal community of tubers in different growth phases and the soils surrounding *G. elata* were characterized by high-throughput sequencing (Chen et al., 2019; Yuan et al., 2020). Mycorrhizosphere bacteria, *Rahnella* sp. HPDA25 has been proven to secrete indole-3-acetic acid to promote the growth of *A. gallica* and its parasitic host *G. elata* (Liu et al., 2022). The coculture of HPDA25 and *A. gallica* also decreased the expression levels of glycolysis-related genes, which may advance rhizomorph growth by inhibiting glycolysis in *A. gallica*. *Irpex lacteus* tended to promote the growth of *Armillaria* in coculture by producing 2,3-dihydroxydodacane-4,7-dione to selectively inhibit the phytopathogen and endophyte in the host *G. elata*, which is conducive to the symbiosis of *G. elata* and *Armillaria* symbiosis (Wang et al., 2021a). Many beneficial compounds were isolated from the coculture of *Armillaria* sp. and the endophytic fungus associated with *G. elata* (Li et al., 2019; Li et al., 2020a). In contrast, *Armillaria* can also affect the structure of the microbial community associated with *G. elata*, as evidenced by the increased diversity of bacteria and fungi from the immature tuber to mature tuber periods (Yuan et al., 2018). In addition, early studies have shown that *Armillaria*, as a medicinal fungus, can secrete various antibacterial and antifungal compounds, such as armillaric acid and sesquiterpene aryl esters, which show strong inhibition against gram-positive bacteria, *Streptococcus* spp., yeast, *Rifai aggr.*, *Mucor* spp., *Gliocladium viren*, *Fusarium* spp., *Rhizopus stoloniferp*, and *Trichoderma* spp. (Yuan et al., 2020). *Mycena* is a source of plant hormones and nutrients for *G. elata* according to a study of the secondary metabolites of fungi (Liang et al., 2018).

## 5 Conclusion and future perspectives

The roles that *Mycena* and *Armillaria* play in the life cycle of *G. elata* are crucial. Various developmental phases result in various histological and ultrastructural properties during the colonization and digestion of hyphae in *G. elata*. The material basis of symbiotic partnerships is the bilateral flow of nutrients between *G. elata* and fungi. Moreover, to adapt to symbiosis, gene expression and enzyme release are altered in both fungi and *G. elata*. Additionally, some microbes and their byproducts are advantageous for symbiosis between fungi and *G. elata*. We have thoroughly explained the three components of the symbiotic mechanism: morphology, feeding, and chemicals.

However, there are still many issues with the symbiotic mechanism that need to be further clarified. We believe that the following topics should be the main focus of future research. i) What are the advantages of *G. elata* and fungi developing a symbiotic connection in order for both to adapt to the natural environment? ii) What evolutionary trends and features do fungi and *G. elata* have in their genomes that enable them to develop and sustain symbiotic relationships? iii) How are the immune systems, signaling pathways, and metabolic processes of fungi and *G. elata* regulated and controlled to adapt to symbiosis?

## Author contributions

J-JL: Writing – original draft. X-QY: Visualization, Writing – original draft. Z-YL: Investigation, Visualization, Writing – original draft. J-YM: Investigation, Resources, Writing – original draft. S-BL: Investigation, Visualization, Writing – original draft. W-PZ: Writing – review & editing. Y-CL: Funding acquisition, Writing – review & editing. L-BL: Funding acquisition, Project administration, Supervision, Writing – review & editing.
